# Development and validation of a multivariable model for prediction of malignant transformation and recurrence of oral epithelial dysplasia

**DOI:** 10.1038/s41416-023-02438-0

**Published:** 2023-09-27

**Authors:** Hanya Mahmood, Adam Shephard, Paul Hankinson, Mike Bradburn, Anna Luiza Damaceno Araujo, Alan Roger Santos-Silva, Marcio Ajudarte Lopes, Pablo Agustin Vargas, Kris D. McCombe, Stephanie G. Craig, Jacqueline James, Jill Brooks, Paul Nankivell, Hisham Mehanna, Nasir Rajpoot, Syed Ali Khurram

**Affiliations:** 1 Academic Unit of Oral & Maxillofacial Surgery, School of Clinical Dentistry, University of Sheffield, Sheffield, UK; 2 Tissue Image Analytics Centre, Department of Computer Science, University of Warwick, Warwick, UK; 3 Unit of Oral & Maxillofacial Pathology, School of Clinical Dentistry, University of Sheffield, Sheffield, UK; 4 Clinical Trials Research Unit, School of Health and Related Research, University of Sheffield, Sheffield, UK; 5 Oral Diagnosis Department, Piracicaba Dental School, University of Campinas (UNICAMP), São Paulo, Brazil; 6 Precision Medicine Centre, Patrick G. Johnston Centre for Cancer Research, Queen’s University Belfast, Belfast, UK; 7 Institute of Head and Neck Studies and Education, Institute of Cancer and Genomic Sciences, University of Birmingham, Birmingham, UK

**Keywords:** Oral cancer, Oral cancer

## Abstract

**Background:**

Oral epithelial dysplasia (OED) is the precursor to oral squamous cell carcinoma which is amongst the top ten cancers worldwide. Prognostic significance of conventional histological features in OED is not well established. Many additional histological abnormalities are seen in OED, but are insufficiently investigated, and have not been correlated to clinical outcomes.

**Methods:**

A digital quantitative analysis of epithelial cellularity, nuclear geometry, cytoplasm staining intensity and epithelial architecture/thickness is conducted on 75 OED whole-slide images (252 regions of interest) with feature-specific comparisons between grades and against non-dysplastic/control cases. Multivariable models were developed to evaluate prediction of OED recurrence and malignant transformation. The best performing models were externally validated on unseen cases pooled from four different centres (*n* = 121), of which 32% progressed to cancer, with an average transformation time of 45 months.

**Results:**

Grade-based differences were seen for cytoplasmic eosin, nuclear eccentricity, and circularity in basal epithelial cells of OED (*p* < 0.05). Nucleus circularity was associated with OED recurrence (*p* = 0.018) and epithelial perimeter associated with malignant transformation (*p* = 0.03). The developed model demonstrated superior predictive potential for malignant transformation (AUROC 0.77) and OED recurrence (AUROC 0.74) as compared with conventional WHO grading (AUROC 0.68 and 0.71, respectively). External validation supported the prognostic strength of this model.

**Conclusions:**

This study supports a novel prognostic model which outperforms existing grading systems. Further studies are warranted to evaluate its significance for OED prognostication.

## Background

Oral epithelial dysplasia (OED) is a ‘pre-cancerous state’ histologically characterised by cellular atypia with loss of normal maturation and stratification of stratified squamous epithelium [1, 2]. Its progression to malignancy (oral squamous cell carcinoma or OSCC) is a progressive multi-step process which can be initiated by chemical carcinogen exposure (such as tobacco) [3], genetic mutations [4–6] and in a small subset of cases, by high-risk human papilloma virus (HPV) [7]. The progression of OED to OSCC is variable (mild OED 1.7%; severe OED 3.57%, annually) [8, 9] and difficult to predict due to poor understanding of the disease pathway [10, 11].

Conventionally, a diagnosis of OED is reached following identification of a wide range of histological architectural (whole epithelium) and cytological (individual keratinocyte) abnormalities using the World Health Organisation (WHO) criteria [12]. This three-tier grading system (mild, moderate, severe) was recently updated from the fifth edition of the WHO classification to further expand the range of diagnostic features to twenty-seven in total (14). However, the prognostic strength of these features remains poorly understood [13, 11] and, individually, many of them are relatively non-specific [11] and evident in a host of other non-dysplastic conditions (such as reactive atypia in inflammatory and ulcerative conditions or fungal infections) [11, 14]. As such, conventional grading is an unreliable predictor of cancer risk, further complicated by inter and intra-observer inconsistencies [15], variations in interpretation of findings [11], and alternative proposed grading systems [16, 17]. Grading should, therefore, not be used as a sole indicator for treatment selection.

In addition to the ‘conventional’ OED features, there are also a range of other features seen in OED, which are not routinely quantified or analysed by the pathologist, nor known to be correlated to clinical outcomes. Such features include alteration in cell numbers, differences in lesion architecture and thickness, variations in nuclei geometry and staining intensity of cell cytoplasm. The importance of these features in OED progression to malignancy has not been given much attention, perhaps due to difficulty in their visual assessment using conventional microscopy methods, and the time consuming and laborious nature of cellular level analysis.

Whilst several studies have focussed on the strength of grading alone, it is important to acknowledge that the ‘global’ grade is not always representative of feature severity, nor does it consider clinical variables (such as age, gender or clinical site). More recently, the ‘six-point’ and ‘two-point’ prognostic models were developed using cytological and architectures features associated with malignant transformation and recurrence with good inter-observer agreement [18]. The authors found that the strength of these models increased when combined with histological grading and clinical characteristics, outperforming conventional grading systems alone. This highlights the need to further explore prognostic associations of novel histological variables in a similar manner, through development and testing of multivariable models. With the advancement of digital pathology methods, it is now possible to conduct detailed quantitative histological analyses of digitised whole-slide images (or WSI) of Haematoxylin and Eosin (H&E) stained tissue sections using computer-assisted approaches [19]. WSIs contain large volumes of data which can be useful for exploration of prognostic markers. Digital image analysis allows automated detection of cell nuclei and subsequent quantification assessment of subcellular compartments [20] generating data in an objective and reproducible manner for downstream analysis.

This study consists of three parts. First, we conduct a digital quantitative analysis of cellularity, nuclei morphometry, cell cytoplasm colour intensity and thickness/perimeter in OED epithelium, to explore differences between dysplasia grades and non-dysplastic oral epithelium. Secondly, we explore the prognostic value of these features and develop a predictive model for OED recurrence and malignant progression. Finally, we conduct external validation of the proposed model using three independent datasets from other national and international centres.

Whilst the application of digital image analysis to study oral premalignant disorders is increasing, few studies have applied these methods for exploration of histological predictors in OED. This study provides novel insight into OED progression, identifying new and potentially important features for clinical outcome prediction.

## Materials and methods

### Training dataset and clinical data

A retrospective sample of 75 OED cases (one representative H&E slide per case) were used for quantitative histological feature analysis and development of the multivariate predictive models. Where feature-specific comparisons are made, a control sample of 25 non-dysplastic oral tissue sections (including hyperplasia and traumatic hyperkeratosis) were used. These slides were obtained from the local pathology archive (School of Clinical Dentistry, University of Sheffield, UK, dating 2008–2013). Purposive sampling was used to include equal numbers for mild, moderate and severe grades and controls (*n* = 25 each). A minimum of 5-year clinical follow-up was required. Cases were also re-graded using the binary OED grading system.

Prior to the inclusion of cases, slides were independently reviewed by two pathologists (SAK, PH) to ensure tissue sections were of suitable quality for analysis. HPV-related OED and verrucous lesions were excluded, based on morphological analysis, as they are distinct entities with reportedly different features and behaviour. Cases were also excluded if (1) there was no associated H&E slide, (2) there was insufficient epithelial tissue for analysis, (3) the slide was of poor staining quality, appeared distorted/blurred, had tissue artefacts/folds or (4) there was incomplete or irretrievable follow-up data. Ethical approval was obtained (18/WM/0335) and experimental methods were conducted in line with the Declaration of Helsinki.

Clinical data were obtained from patient case notes and various online hospital clinical systems. Collected data included demographic details and relevant diagnostic information including intra-oral biopsy site, original histological OED grade (WHO, 2017), treatment information, and whether the lesion had transformed or recurred. The clinicians abstracting this data were blinded to patient outcomes. We defined transformation as a dysplastic lesion which had progressed to OSCC at the same clinical site, and recurrence as a dysplastic lesion which had occurred at the same clinical site following surgical excision within the follow-up period.

### Quantitative histological feature analysis

New 4 µm H&E sections of the selected cases were digitised to high-resolution WSIs using Aperio CS2 (Leica Biosystems, Germany) and Hamamatsu NanoZoomer 360 (Japan) scanners. QuPath (version 0.3.0) bioimage analysis software [21] was used for quantitative histological analysis. This platform was chosen due to its powerful annotation, visualisation and built-in cell and nuclear detection tools [21], in addition to its reported reproducibility in tissue-based biomarker studies [22].

Regions of interest (ROIs) corresponding to histologically representative areas were identified for each image and confirmed by several authors (HM, PH, SAK). Within each ROI, the full thickness of the epithelium was demarcated for localised quantitative analysis. For consistency, a minimum of three and a maximum of four ROIs were selected per whole-slide image, with a closely matched area (300,000 ± 400 µm^^2^). A standardised cell detection threshold of 0.04 was set (at ×10 magnification) and other default parameters were kept consistent across all ROIs. The cell detection algorithm in QuPath was utilised to quantify cell numbers and extract a range of other features as outlined below:

i. Number of epithelial cells
ii. Nuclear geometry: nuclear circularity, nuclear eccentricity, nucleus to cell area ratio
iii. Staining intensity: nuclear haematoxylin optical density (OD), cytoplasm eosin OD
iv. Epithelium architecture/thickness: Perimeter (µm)

Due to the inherent nature of OED, ROI-level analysis was considered better than WSI-level analysis, as dysplasia is not always visualised throughout the tissue section. In total, 325 ROIs were generated for analysis and model development (mild OED *n* = 84, moderate OED *n* = 83, severe OED *n* = 85, control *n* = 73). Extracted data were systematically recorded in an Excel spreadsheet (Microsoft Corporation, 2018).

### Measuring cell detection accuracy

**Fig. 1:**
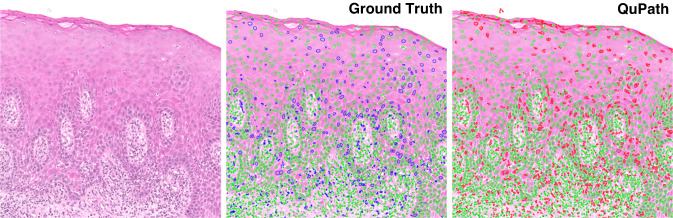
A comparison of the ground truth (middle) vs. QuPath’s cell detection tool (right) for nuclear detection and segmentation. The raw image is displayed on the left. Colour key: green nuclei = true positives; blue nuclei = false negatives; red nuclei = false positives.

To assess the reliability of QuPath’s cell detection algorithm, we conducted a measure of accuracy based on 10 ROIs (Fig. 1). Ground-truth nuclear segmentations within these 10 ROIs were generated semi-automatically. An expert oral pathologist (SAK) clicked on each nucleus in the 10 ROIs. These nuclear ‘clicks’ were then passed through NuClick [23], a deep learning model that takes ‘click’ inputs as a guiding signal to generate nuclear boundaries. These segmentations were then further refined manually, where necessary, to ensure accurate nuclear segmentations. On comparison of the QuPath nuclear segmentations to the ground-truth segmentations, the results were promising, producing a raw Dice score of 0.73 (nuclei vs. background), a detection quality score of 0.67, a segmentation quality score of 0.72 and a panoptic quality of 0.49. These scores are in line with more recent nuclear instance segmentation papers such as HoVer-Net [24], HoVer-Net+ [25] and PanNuke [26], where Dice score and segmentation quality measure the performance of nuclei segmentation and detection quality, panoptic quality and aggregated jaccard index give measures of the individual nuclear detections.

### Development and validation of prognostic models

Multivariable logistic regression analysis was conducted to model the different histological features for prediction of malignant transformation and OED recurrence. The models were developed using different combinations of parameters, and for clinical interest we did not restrict these combinations to only features that were statistically significant. Model performance was visualised by measuring the Area Under the Receiver Operating Characteristic (AUROC) curve.

External validation was performed using further OED cases pooled from four different centres (*n* = 121, 287 ROI: 79 mild, 106 moderate and 112 severe) (Table 1); these cases were not part of the original model development. 60 patients were female (49.5%) and 61 were male (50.5%), with a mean age of 59 years (S.D 12.55). Intra-oral sites included ventral/lateral tongue (60%, *n* = 72), buccal mucosa (14%, *n* = 17), floor of mouth (16%, *n* = 20), palate (6%, *n* = 7) and alveolar ridge (4%, *n* = 5). Independent grading confirmed 27% mild (*n* = 33), 36% moderate (*n* = 43) and 37% severe (*n* = 45) OED lesions. Binary grading confirmed 43 low grade (36%) and 78 high grade (64%) lesions. 39 lesions progressed to OSCC (32%), with an average time to transformation of 45 months (median 42, S.D 33, IQR 54) (Table 1). Amongst the 121 cases, only 34 cases were used for validation of the recurrence model; the remainder could not be used to reliably predict recurrence status due to incomplete follow-up data. The average time to recurrence was 19 months (median 12, S.D 17, IQR 28).

**Table 1:** Cohorts for model development and validation with respective grade (WHO, 2017) breakdown and clinical outcomes.

	*N*	Transformation	Recurrence
DEVELOPMENT COHORT			
School of Clinical Dentistry, Sheffield, UK			
Mild	25	1 (1%)	2 (3%)
Moderate	25	9 (12%	11 (15%)
Severe	25	9 (12%)	13 (17%)
* Total*	*75*	*19 (25%)*	*26 (35%)*
*Overall Total*	***75***		

VALIDATION COHORT			
School of Clinical Dentistry, Sheffield, UK			
Mild	4	0 (0%)	0 (0%)
Moderate	6	2 (13%)	2 (13%)
Severe	5	3 (20%)	2 (13%)
* Total*	*15*	*5 (33%)*	*4 (27%)*
Piracicaba Dental School, UNICAMP, Brazil			
Mild	8	2 (11%)	1 (5%)
Moderate	7	1 (5%)	1 (5%)
Severe	4	1 (5%)	1 (5%)
* Total*	*19*	*4 (21%)*	*3 (15%)*
Queen’s University Belfast, UK			
Mild	1	1 (2%)	Not recorded
Moderate	16	10 (22%)	
Severe	28	14 (31%)	
* Total*	*45*	*25 (55%)*	
Institute of Head and Neck Studies and Education (InHANSE), Birmingham, UK			
Mild	20	0 (0%)	Not recorded
Moderate	14	3 (7%)	
Severe	8	2 (5%)	
* Total*	*42*	*5 (12%)*	
*Overall Total*	***121***		

The total number in the development and validation cohort are in bold.

### Statistical analysis

GraphPad Prism® statistical software (version 9.3.1) was used for analysis. Descriptive statistics were performed for all histological variables. Continuous data were tested for normality using Shapiro–Wilk or D'Agostino & Pearson tests. Where normal distribution was assumed, an unpaired two-tailed *T*-test or one-way ANOVA with an applicable post-hoc analysis (Tukey’s or Dunnett’s) was performed for pairwise comparisons. Individual histological feature associations with clinical outcomes were determined using binary logistic regression, and multivariate regression analysis for the development and testing of the models. Prognostic discrimination was visualised by AUROC curves with a 95% confidence interval. All tests were two-tailed, and *p* values adjusted for multiple comparisons testing.

## Results

### Quantitative analysis and model development

Amongst the OED dataset used for quantitative feature analysis and model development (*n* = 75), independent grading assessment confirmed 25 each for mild, moderate and severe WHO grades (Table 1). Binary grading revealed 33 low grade and 42 high grade lesions. 47 patients (63%) were male and 28 (37%) females with a median age of 65 years (IQR 21). Intra-oral sites included the floor of mouth (*n* = 15, 20%), buccal/labial mucosa (*n* = 13, 17%), tongue (*n* = 39, 52%), gingivae (*n* = 4, 5%) and hard/soft palate (*n* = 4, 5%). 25% of lesions (*n* = 19) progressed to OSCC, with an average transformation time of 31 months (median 24, S.D 26.14, IQR 48). 35% of lesions recurred (*n* = 26) with an average recurrence time of 35 months (median 24, S.D 35.42, IQR 24) (Table 1).

### Quantitative analysis of cellularity

**Fig. 2:**
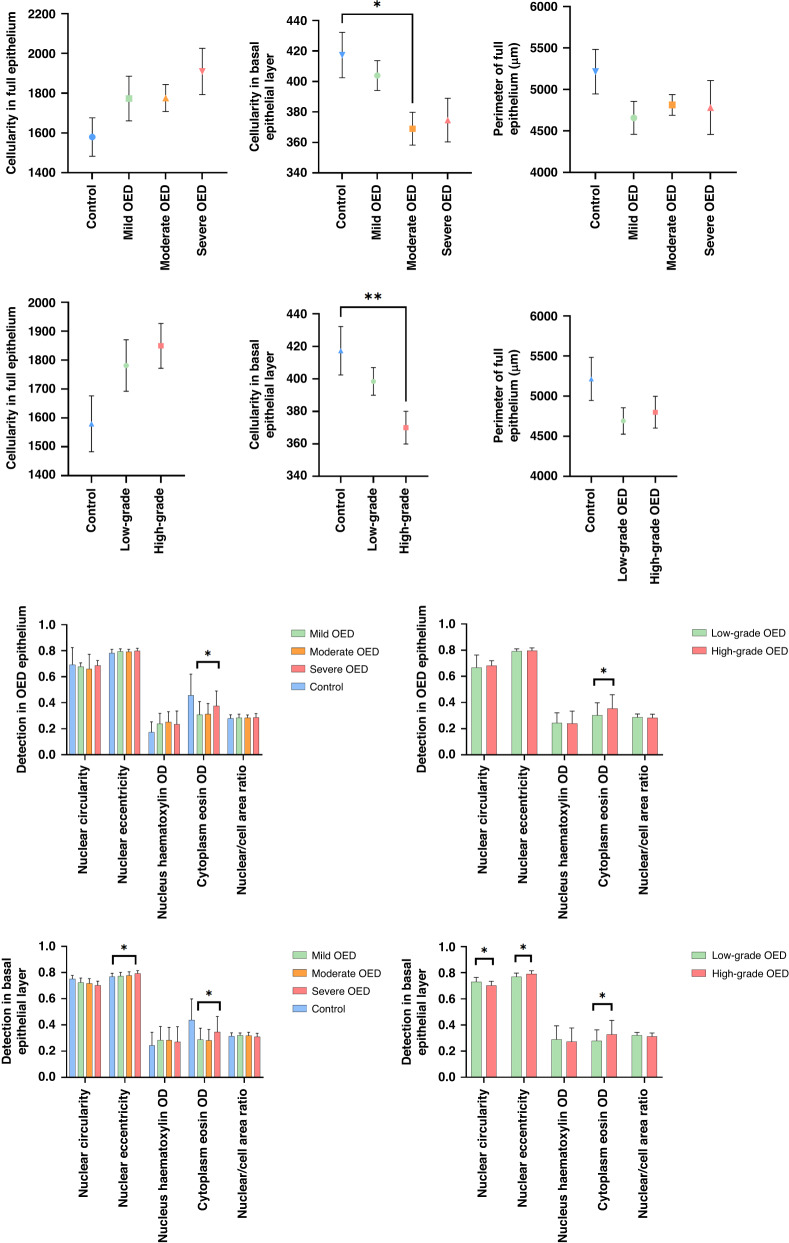
Grade-wise analysis of cellularity, nuclear and cytoplasmic features and perimeter in OED with comparison to control. Displayed values represent mean values ± standard error. Asterisk denotes statistically significant result (* *p* ≤ 0.05, ** *p* ≤ 0.01).

Increased epithelial cellularity was seen in OED compared to control (mean cell number: mild OED 1773 [95% CI 1541–2005], moderate OED 1776 [95% CI 1637–1915], severe OED 1909 [95% CI 1600–2076] vs. control 1508 [95% CI 1302–1728]), though these differences were not significant (Fig. 2). In contrast, the cellularity in OED was reduced in the basal epithelial layer of OED compared to control (mild OED + moderate OED + severe OED vs. control *p* = 0.02; low grade OED + high grade OED vs. control *p* = 0.01). Further differences were observed between individual grades: moderate OED vs. control (*p* = 0.02, 95% CI 5.66–90.99) and high grade OED vs. control (*p* = 0.007, 95% CI 11.75–82.87) (Fig. 2). There was no statistical association between epithelial cellularity (in the full thickness of the epithelium or basal epithelial layer) with clinical outcomes (Table 2).

**Table 2:** Statistical association of individual histological features with clinical outcomes for the full thickness of the epithelium and basal epithelial layer (*n* = 75).

	Full epithelium		Full epithelium		Basal epithelial layer		Basal epithelial layer	
Event	MT		R		MT		R	
	AUROC	*p* value	AUROC	*p* value	AUROC	*p* value	AUROC	*p* value
Cellularity	0.5075	0.7877	0.5188	0.841	0.5241	0.7824	0.5137	0.6818
Perimeter	0.6118	0.0336*	0.5683	0.1012	*not assessed*			
Nucleus circularity	0.6471	0.0645	0.6903	0.0180*	0.535	0.7659	0.5534	0.6668
Nucleus eccentricity	0.6405	0.0639	0.5687	0.2975	0.5145	0.9563	0.5573	0.2392
Nucleus haematoxylin OD	0.5348	0.8844	0.5553	0.5495	0.5464	0.6162	0.5522	0.5059
Cytoplasm eosin OD	0.5531	0.4115	0.5856	0.1747	0.5559	0.5787	0.5895	0.1915
Nuclear/cell area ratio	0.5428	0.2621	0.5114	0.6103	0.5423	0.3326	0.5353	0.3967

Asterisk denotes statistically significant result (* *p* ≤ 0.05, ** *p* ≤ 0.01).

*MT* malignant transformation, *R* OED recurrence *AUROC* area under receiver operating characteristic.

### Quantitative analysis of nuclear and cytoplasmic features

Grade-related differences in cytoplasmic eosin OD were seen in OED epithelium (mild OED vs. moderate OED vs. severe OED *p* = 0.03 and low grade vs. high grade OED *p* = 0.02) with higher detection levels in the more severe lesions (Fig. 2). No significant grade-related differences were seen for nuclear circularity, nuclear eccentricity, nuclear haematoxylin OD or nucleus/cell area ratio (Fig. 2). Basal epithelial layer analysis demonstrated significant differences between WHO grades for nuclear eccentricity (*p* = 0.02) and cytoplasm eosin (*p* = 0.04). These two features were also significant between binary grades (*p* = 0.0004 and *p* = 0.04, respectively) in addition to nuclear circularity (*p* = 0.0005) (Fig. 2). There was no statistical association between individual nuclear and cytoplasmic features (for either full epithelium or basal epithelial layer) and malignant progression. However, nuclear circularity in the full epithelium was associated with OED recurrence (*p* = 0.02, AUROC 0.69, 95% CI 0.56–0.82) (Table 2).

### Quantitative analysis of thickness/perimeter of OED epithelium

There were no significant differences in the perimeter/thickness of the full epithelium between OED grades (WHO or binary) or comparison to controls (Fig. 2). However, there was a statistical association between the perimeter of OED epithelium and malignant transformation (*p* = 0.02, AUROC 0.61 with 95% CI 0.47–0.75) (Table 2).

### Prediction of malignant transformation

**Fig. 3:**
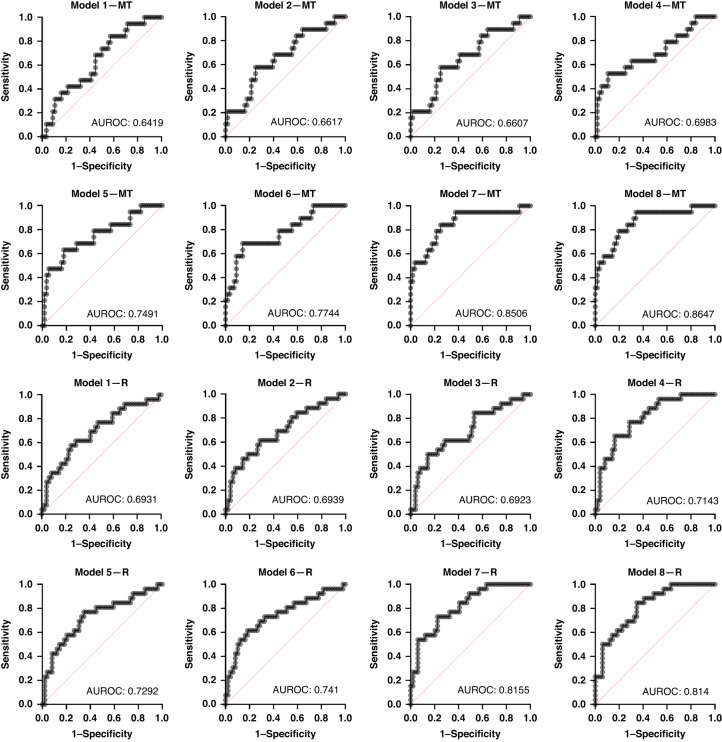
ROC curves for multivariate models for malignant transformation (MT) and OED recurrence (R).

Comparisons between the various models demonstrated that increasing the number of histological variables strengthened the models’ predictive performance, both for malignant transformation and OED recurrence (Table 3). Model 6 (“epithelial cellularity” + “nuclear circularity” + “nuclear eccentricity” + “nucleus haematoxylin OD mean” + “cytoplasm eosin OD mean” + “nuclear/cell area ratio” + “perimeter of epithelium”) showed good prognostic value for prediction of malignant transformation (AUROC 0.77, 95% CI 0.65–0.90, *p* = 0.0004) compared to models with fewer histological variables (Table 3, Fig. 3). This model demonstrated a negative predictive power of 81.25% and a positive predictive power of 63.64%. The odds ratios for individual features formulating this model are presented in Supplementary Table 1. The strength of this model was further improved by incorporating histological grading systems. The addition of “WHO grading” (model 7) increased AUROC to 0.85 (95% CI 0.74–0.96, *p* <0.0001), and the addition of “Binary grading” (model 8) produced an AUROC of 0.86 (95% CI 0.77–0.96, *p* < 0.0001) (Table 3, Fig. 3). These models performed better than WHO grading (AUROC 0.68, 95% CI 0.56–0.81, *p* = 0.014) and binary grading systems alone (AUROC 0.72, 95% CI 0.60–0.84, *p* = 0.003) for prediction of malignancy.

**Table 3:** Multivariate histological models for prediction of malignant transformation (MT) and OED recurrence (R).

Each model is developed using 75 OED slides (252 ROI; 84 mild, 83 moderate, 85 severe). The first two rows indicate the prognostic strength of individual grading systems without additional variables, for reference. The rows highlighted in grey indicate the external validation results for model prediction (MT, *n* = 121; R, *n* = 34).

^a^Denotes a statistically significant finding.

External validation was conducted on 121 additional cases using the same features as in model 6. Doing so showed a similar AUROC of 0.76 (95% CI 0.68–0.85, *p* < 0.0001) for malignant transformation (Table 3).

### Prediction of OED recurrence

Model 6 (“epithelial cellularity” + “nuclear circularity” + “nuclear eccentricity” + “nucleus haematoxylin OD mean” + “cytoplasm eosin OD mean” + “nuclear/cell area ratio” + “perimeter of epithelium”) also demonstrated good performance for prediction of OED recurrence with an AUROC of 0.74 (95% CI 0.62–0.87, *p* = 0.0006) in comparison to models 1–5 where AUROC ranged between 0.69 and 0.72 (Table 3, Fig. 3). This model demonstrated a negative predictive power of 74.58% and a positive predictive power of 68.75%. The odds ratio for individual features formulating this model are presented in Supplementary Table 2. Similar to the malignant transformation models, the incorporation of current grading systems increased model performance. The addition of “Binary grading” (model 8) increased AUROC to 0.81 (95% CI 0.72–0.91, *p* < 0.0001), and addition of “WHO grading” (model 7) optimised the performance further, yielding an AUROC of 0.82 (95% CI 0.72–0.91, *p* < 0.0001) (Table 3, Fig. 3).

External validation was limited to 34 individuals with confirmed recurrence status. Model 6 retained its superior performance with an AUROC of 0.93 (95% CI 0.81–1.00, *p* = 0.0005) for OED recurrence (Table 3).

## Discussion

In this study, we use digital image analysis to explore and extract quantitative data for several histological features (cell number, nuclear and cytoplasm geometric and intensity features, lesion thickness/perimeter) in OED epithelium to determine their diagnostic importance and relationship with clinical outcomes. We focussed the analysis on the full thickness of the epithelium, as opposed to conventional epithelial ‘thirds’ used with WHO grading, to remove layer restriction and subjectivity.

The unique aspect of this study is the development of multivariate models for outcome prediction using the digitally quantified histological data. Our findings demonstrated that the combination of all the major digital histological features (Model 6: “epithelial cellularity” + “nuclear circularity” + “nuclear eccentricity” + “nucleus haematoxylin OD mean” + “cytoplasm eosin OD mean” + “nuclear/cell area ratio” + “perimeter of epithelium”) was associated with greater predictive performance for both malignant transformation and OED recurrence in comparison to conventional histological grading systems (Table 3, Fig. 3). Model 6 yielded good predictive performance for malignant transformation (AUROC of 0.77, 95% CI 0.64–0.90, *p* = 0.0004) and OED recurrence (AUROC of 0.74, 95% CI 0.61–0.86, *p* = 0.0006) which exceeded that of WHO grading (AUROC 0.69, *p* = 0.01) and binary grading (AUROC 0.72, *p* = 0.0037) alone. External validation of model 6 supported its superior performance (AUROC of 0.76, 95% CI 0.68 –0.85, *p* < 0.0001 for malignant transformation and AUROC of 0.93, 95% CI 0.81–1.00, *p* = 0.0005 for OED recurrence) (Table 3). The performance of this model was further enhanced by adding individual grading systems (Models 7 and 8, Table 3), which yielded even better predictive potential than each alone, highlighting the potentially valid contribution of grading to clinical outcome prediction. More extensive validation on larger datasets is needed to establish the clinical utility of these features and models.

With regards to epithelial cellularity, there was an increased cell number in OED epithelium (compared to control) with more pronounced cellularity in severe/high grade OED lesions (Fig. 2). In contrast, a reduced basal cellularity was seen in OED epithelium compared to control (*p* = 0.02) with similar differences between moderate OED vs. control (*p* = 0.02) and high grade OED vs. control (*p* = 0.007). This is contrary to what we had expected, since basal cell crowding is thought to be associated with dysplasia severity. This finding can be explained by the increased level of cellular disarrangement and pleomorphism seen in more dysplastic regions, which in turn, may have resulted in fewer cells being detected. Whilst there were no significant prognostic correlations for cellularity in our study, further investigation of its diagnostic importance is worth exploring, considering the quantitative differences observed against non-dysplastic lesions.

Epithelial thickness was quantitatively evaluated by measuring the perimeter (length/distance) of the lesion margin/periphery, as an indirect measure of rete process/ridge morphology, a common feature of OED. Findings demonstrated the perimeter of OED epithelium to be particularly associated with malignant transformation (*p* = 0.03, AUROC 0.61 with 95% CI 0.47–0.75) (Table 2) indicating that epithelial thickness (or indirectly, curvature) may be a potentially important predictor of OED progression.

Whilst some studies have explored the effect of histomorphological characteristics, such as lesion thickness and cellularity in the diagnosis of premalignant lesions, few have studied this specifically in OED. One study showed differences in maximum lesion thickness and cellularity in high grade cervical squamous intraepithelial lesions compared to p16-positive cervical tissue biopsies [27]. In another study, microscopic analysis of oesophageal squamous dysplasia showed increased cellularity, disordered cell arrangement and loss of polarity in the basal layer [28]. Further analysis of keratin thickness, pattern and morphology in OED would be interesting to explore, particularly as abrupt orthokeratosis and verrucous surface architectures are frequently seen in oral potentially malignant disorders.

Analysis of nuclear and cytoplasmic features highlighted certain features to be more pronounced in OED with some differences between grades (Fig. 2). Relevant features include cytoplasm eosin OD (full epithelium, *p* = 0.025–0.035; basal layer, *p* = 0.037–0.039), nucleus eccentricity (basal layer, *p* = 0.0004 for binary grades, *p* = 0.016 for WHO grades) and nucleus circularity (basal layer, *p* = 0.0005 for binary grades). Eosin is a common synthetic dye used in H&E tissue analysis. It is a negatively charged dye which stains basic components of a cell, mainly positively charged proteins (or acidophilic) structures such as amino groups in the cytoplasm a bright pink colour, which contrasts with blue haematoxylin staining [29]. There is a lack of published research to explain the clinical relevance of increased cytoplasm eosin levels, particularly concerning OED diagnosis. However, our findings may be explained by the altered nuclear morphometry in dysplastic cells, which in turn, could affect eosin amount and representation. For example, an increase in cell size (cellular pleomorphism) may relate to increased cytoplasm eosin content. Furthermore, the presence of dyskeratosis and premature/individual cell keratinisation may contribute, giving the cytoplasm a more eosinophilic cytoplasm. Other potentially relevant clinical features relate to nuclear eccentricity (the displacement of the nucleus from the centre of the cell) and nuclear circularity (the degree to which the nucleus has deviated from circularity). Both these features are of diagnostic relevance in dysplastic lesions, in addition to other nuclear morphological features such as circularity, compactness, density and nuclear-cell ratio [30, 31]. In our study, nuclear circularity was also found to be associated with OED recurrence (*p* = 0.02, AUROC 0.693 with 95% CI 0.56–0.82) (Table 2).

The authors acknowledge some limitations of the current study. The first relates to the training sample used for quantitative analysis and model development, which includes cases from a single centre. However, the department in question is a UK national referral centre receiving OED cases from a wide geographical region, thereby incorporating varied samples from different patient groups. Whilst the sample size may be considered small, it must be highlighted that the digital quantitative evaluation has been conducted on 252 OED ROIs which is considered sufficient to draw initial conclusions. The second limitation relates to the selection of cases through purposive sampling, which may introduce an element of bias. However, we have tried to mitigate this risk, as our models have been tested on WSIs from four different national and international centres. These cases include a variable mix of dysplasia grades, a large proportion of transformed cases and have been scanned using different scanners. As such, the sample has increased biological and technical diversity which improves the robustness and generalisability of the developed models. Another limitation relates to potential staining variations between WSI cohorts. This has been somewhat overcome by using a training sample from a single centre, which follows consistent staining protocols. Whilst stain variation may introduce problems when machine learning algorithms are being developed, it is less problematic in purely quantitative digital image analysis. Slide stains are also not ‘normalised’ in routine diagnostic practice, so it is a more representative reflection of real-world approaches.

As we progress towards a digital pathology workflow, this study highlights the potential of digital image analysis tools for the study and prognostication of complex oral diseases. Our findings provide novel insights into OED progression, by identifying new histological predictors that are not routinely considered in diagnostic practice. To the best of the authors’ knowledge, this is the first study to quantify and correlate cellularity, geometric, colour and perimeter features in OED to clinical outcomes. Further analysis on larger multicentric cohorts is needed to validate and refine our findings to determine the full prognostic value of the studied features for wider clinical application.

### Supplementary information

Supplementary Table 1

Supplementary Table 2

## Data Availability

All data analysed during this study are included in this published paper. Correspondence and requests for materials should be addressed to Dr HM.
