# Supplementary material for: Development and validation of a multivariable model for prediction of malignant transformation and recurrence of oral epithelial dysplasia
Source: Br J Cancer. 2023 Sep 27;129(10):1599–607. doi: 10.1038/s41416-023-02438-0 (PMC10645879; doi:10.1038/s41416-023-02438-0)
Supplement: Supplementary file 1 — Supplementary Table 1 [file 41416_2023_2438_MOESM1_ESM.docx]

Supplementary Table 1. Odds ratios for individual features that formulate the best performing model for prediction of malignant transformation (Model 6) using the developmental cohort. Values displayed as E+n, in which E (exponent) multiplies the preceding number by 10 to the nth power.

| **Odds ratios** | **Variable** | **Estimate** | **95% CI** |
| --- | --- | --- | --- |
| β0 | Intercept | 7.835E+14 | 3.197e-008 to 8.855e+035 |
| β1 | Epithelial cellularity | 1 | 0.9982 to 1.002 |
| β2 | nuclear circularity | 234.9 | 0.02073 to 6367947467772 |
| β3 | nuclear eccentricity | 4.979E-21 | 1.853e-044 to 12.74 |
| β4 | nucleus haematoxylin OD mean | 1214 | 0.03482 to 113641481 |
| β5 | cytoplasm eosin OD mean | 93.32 | 0.1408 to 133255 |
| β6 | nuclear/cell area ratio | 5.719E-14 | 4.531e-032 to 853.3 |
| β7 | Perimeter µm of epithelium | 1.001 | 1.000 to 1.001 |
