# Supplementary material for: Development and validation of a multivariable model for prediction of malignant transformation and recurrence of oral epithelial dysplasia
Source: Br J Cancer. 2023 Sep 27;129(10):1599–607. doi: 10.1038/s41416-023-02438-0 (PMC10645879; doi:10.1038/s41416-023-02438-0)
Supplement: Supplementary file 2 — Supplementary Table 2 [file 41416_2023_2438_MOESM2_ESM.docx]

Supplementary Table 2. Odds ratios for individual features that formulate the best performing model for prediction of OED recurrence (Model 6) using the developmental cohort. Values displayed as E+n, in which E (exponent) multiplies the preceding number by 10 to the nth power.

| **Odds ratios** | **Variable** | **Estimate** | **95% CI (profile likelihood)** |
| --- | --- | --- | --- |
| β0 | Intercept | 1.077E-07 | 1.149e-028 to 8534610131791 |
| β1 | Epithelial cellularity | 1 | 0.9984 to 1.002 |
| β2 | nuclear circularity | 746129690 | 1.535 to 5.194e+019 |
| β3 | nuclear eccentricity | 2.759 | 2.311e-019 to 2.101e+019 |
| β4 | nucleus haematoxylin OD mean | 7.074 | 0.0007010 to 95779 |
| β5 | cytoplasm eosin OD mean | 19.16 | 0.05206 to 11090 |
| β6 | nuclear/cell area ratio | 0.0000381 | 3.734e-019 to 1144048481 |
| β7 | Perimeter µm of epithelium | 1 | 0.9998 to 1.001 |
